# The influence of alendronate and tooth extraction on the incidence of osteonecrosis of the jaw among osteoporotic subjects

**DOI:** 10.1371/journal.pone.0196419

**Published:** 2018-04-25

**Authors:** Wei-Yih Chiu, Wei-Shiung Yang, Jung-Yien Chien, Jang-Jaer Lee, Keh-Sung Tsai

**Affiliations:** 1 Division of Endocrinology and Metabolism, Department of Internal Medicine, National Taiwan University Hospital and National Taiwan University College of Medicine, Taipei, Taiwan; 2 Department of Internal Medicine, National Taiwan University Hospital and National Taiwan University College of Medicine, Taipei, Taiwan; 3 Department of Laboratory Medicine, National Taiwan University Hospital and National Taiwan University College of Medicine, Taipei, Taiwan; 4 Graduate Institute of Clinical Medicine, College of Medicine, National Taiwan University, Taipei, Taiwan; 5 Division of Oral and Maxillofacial Surgery, Department of Dentistry, National Taiwan University Hospital, Taipei, Taiwan; Boston University Henry M Goldman School of Dental Medicine, UNITED STATES

## Abstract

**Background:**

Although bisphosphonate-related osteonecrosis of the jaw (ONJ) develops mainly after tooth extractions (TEs), the strength of the association between them and how the existence of the disease among bisphosphonate (BP)-treated osteoporotic patients exposed to TE remain uncertain.

**Methods:**

A nationwide retrospective cohort study investigated the influence of alendronate and TE on the development of ONJ.

**Results:**

Incidence of ONJ following long-term alendronate therapy was 262/100,000 person-years, while no event developed in the control group on raloxifene. Overall prevalence of ONJ in osteoporotic subjects receiving alendronate was estimated at 0.34% which rose to 2.16% after TE. Multiple logistic regression analysis, adjusted for the potential confounders, showed TE (adjusted odds ratio, 9.60 [4.33–21.29]), drug duration exceeding 3 years (3.00 [1.33–6.76]), and concomitant rheumatoid arthritis (4.94 [1.64–14.90]) were independent predictors of ONJ.

**Conclusions:**

This article strengthens the relationship between ONJ and BPs. Among osteoporotic patients exposed to alendronate, TE confers a 9.6-fold increased risk for ONJ and it should be performed with caution irrespective of drug duration.

## Introduction

Osteonecrosis of the jaw (ONJ) is a well-known complication in patients administered intravenous bisphosphonates (BPs) to either prevent oncological bone metastasis or to manage hypercalcemia of malignancy especially in breast cancer [1, 2] and multiple myeloma [3] patients. Although osteoporotic patients received doses one-tenth lower, some studies supported the association [4–10] between BP use and a 3.15 to 7.42-fold increase of the risk [4, 9] for ONJ compared to non-BP therapies used for osteoporosis. In addition, earlier studies showed that 52–65% of ONJ cases were precipitated by tooth extractions (TEs) in cancer patients [11–13] and 44–63% in osteoporotic subjects [4, 6, 14, 15]. The event that triggers the development of ONJ in BP-treated patients appears primarily to be dentoalveolar trauma. The animal experiments also supported the rats treated with bisphosphonates developed ONJ-like disease after extraction of teeth [16]. To reduce the risk of ONJ in long-term BP receivers, it is advisable to avoid unnecessary invasive dental procedures. Nevertheless, dentoalveolar surgery is not contraindicated in osteoporotic patients taking oral BPs. The updated 2014 position paper [17] from the American Association of Oral and Maxillofacial Surgeons (AAOMS) recommends interruption of oral medications for at least two months prior to invasive dental procedures in these patients except individuals treated with oral BPs for less than 3–4 years without risk factors because of the extremely low risk of ONJ in this subset of patients. However, reports investigating the strength of the association between dentoalveolar procedures and ONJ occurrence are sparse and studies assessing the AAOMS strategies to reduce ONJ risk before performing invasive dental procedures in bisphosphonate-treated osteoporotic patients remain limited. The aim of this study was to evaluate the relative risks of BP and TE on the development of ONJ and to assess the appropriateness of the preventive approach offered by AAOMS in patients receiving oral BP therapy for osteoporosis using a nationwide longitudinal health insurance research database.

## Materials and methods

### Data source

Data was retrieved from Taiwan’s Longitudinal Health Insurance Database (LHID) covering the period of January 1, 2000 to December 31, 2012. The LHID contains the entire original claim data of 1,000,000 beneficiaries who were randomly sampled in the year 2005 from the records of the 25.68 million people contained in the Taiwan National Health Insurance Research Database (NHIRD). The National Health Insurance program was established in 1995 and insured approximately 99.9% of Taiwan inhabitants by 2014. The LHID research database contains patients’ medical expense claims, ambulatory care, inpatient care, pharmacy data, dates of service, and diagnostic codes based on the International Classification of Diseases, Ninth Revision, Clinical Modification Code (ICD9-CM). There was no significant difference in the age or gender distribution between the patients in the LHID and the original NHIRD (entire research dataset). All of the patients’ identifications have been encrypted to safeguard privacy. The Institutional Review Board at National Taiwan University Hospital approved this study (201405011W) analyzing anonymous secondary data from the LHID and exempt from written informed consent.

### Study design: Cohort identification and outcomes verification

Patients taking oral alendronate for 30 days or longer served as the experimental group and were compared with those patients taking oral raloxifene for 30 days or longer (the control group). Regulations governing the insurance reimbursement of both medications are identical and alendronate and raloxifene were the top two most commonly prescribed osteoporosis drugs in Taiwan during the study period. Considering the mean age of menopause in women and the usual onset of senile osteoporosis in men, women aged 50 years or older or men aged 60 years or older who began taking oral alendronate or raloxifene in the LHID were identified. Subjects were excluded who received denosumab or BPs (except for alendronate) with a drug effect of beyond 30 days within 6 months before the index date. Patients with a diagnostic code for Paget’s disease (ICD9 code 731.0) throughout the study period were also excluded. To identify the ONJ cases, all three criteria modified from the 2014 definitions proposed by the AAOMS [17] were required: (1) After the index date, two or more mentions using an ICD-9 potential code for ONJ (S1 Table) from a dentist appearing in separate encounters and duration of time with such an encounter diagnosis longer than eight weeks; (2) one dental operation for management of ONJ (S2 Table) was performed following the codes for ONJ made by dentist; (3) no head and neck cancers (ICD9 codes 140–149, 160, and 161) was identified before or during the treatment period of antiresorptive therapy (in order to exclude individuals with history of radiation therapy or metastasis to the jaw).

All patients were followed-up until the timing of first dental operation to treat ONJ, a switch from alendronate to other BP or denosumab, one year after cessation of anti-resorptive therapy, permanent disenrollment from the national health insurance, or the end of the study (December 31, 2012), whichever came first. Health care order databases were checked retrospectively to determine whether tooth extraction procedures (including extractions and odontectomy termed either “simple” or “complicated”) had been performed during the follow-up period. To compare the clinical characteristics between exposed and non-exposed groups/factors, medical conditions (such as hypertension or hyperlipidemia) in the prior year of index dates or within 1 year before the endpoints were ascertained with verification of disease status using health care order databases. In the same way, the status of TE was dependent on whether dental extraction procedures had been performed in the prior year of the index date or within one year before the end date in all participants. Health care pharmacy databases were used to determine time-varying exposures of the bisphosphonate, glucocorticoid therapy or methotrexate for each participant, using dispensing date, dosage and frequency. The days over which the medication was dispensed were calculated by dividing the amount of the medication used in 1 day by the dispensed amount. The duration of drug exposure for each subject was calculated as the sum of days the medication was dispensed from the start date to the end date. Chronic steroid use was defined as equivalent to 5 mg or more of prednisone daily for 3 months or longer during the past year; likewise, chronic methotrexate use was specified as greater than or equal to 7.5 mg a week for more than 3 months.

### Statistical analysis

Crude incidence was calculated as the numbers of ascertained ONJ cases divided by person-years involved. To compare baseline characteristics in subjects taking alendronate with and without ONJ, categorical variables were expressed as counts and percentages and compared using Fisher’s exact test. Continuous variables were reported as means ± standard deviations (SD) (with 95% confidence interval [CI] where appropriate) and compared by 2-sided Student’s *t* tests with unequal variances. Multiple logistic regression analysis was used to adjust the specific risk that each independent determinant attributed to the dependent variable. The Kaplan-Meier method was used to estimate the median interval to ONJ occurrence between alendronate and raloxifene groups. The significance level was defined as 0.05. Statistical analysis was performed using the Stata version 11.2 statistical software package (StataCorp, College Station, Texas, US).

## Results

### Alendronate was associated with a higher incidence of ONJ in osteoporotic patients

**Fig 1:**
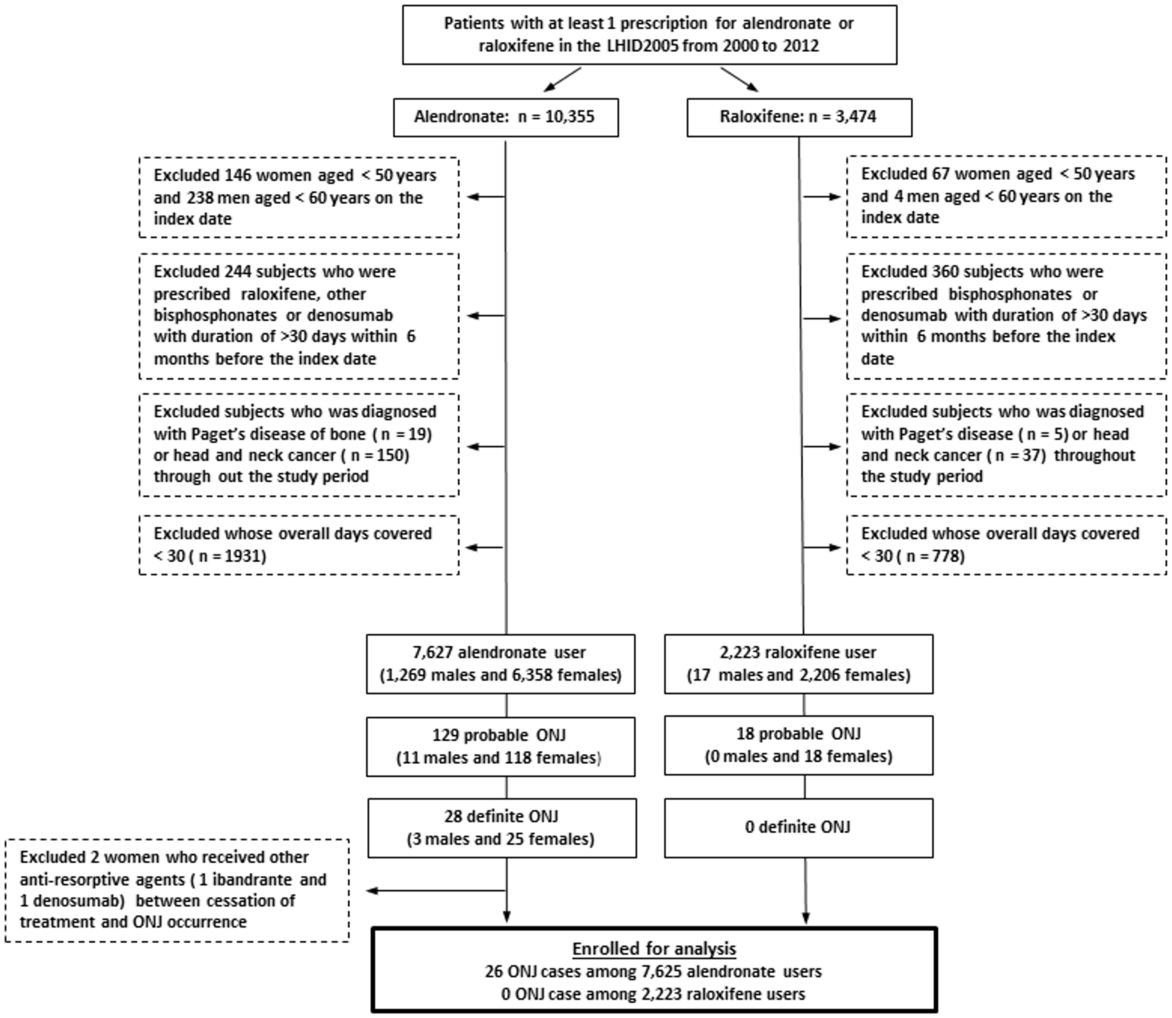
Study design.

**Fig 2:**
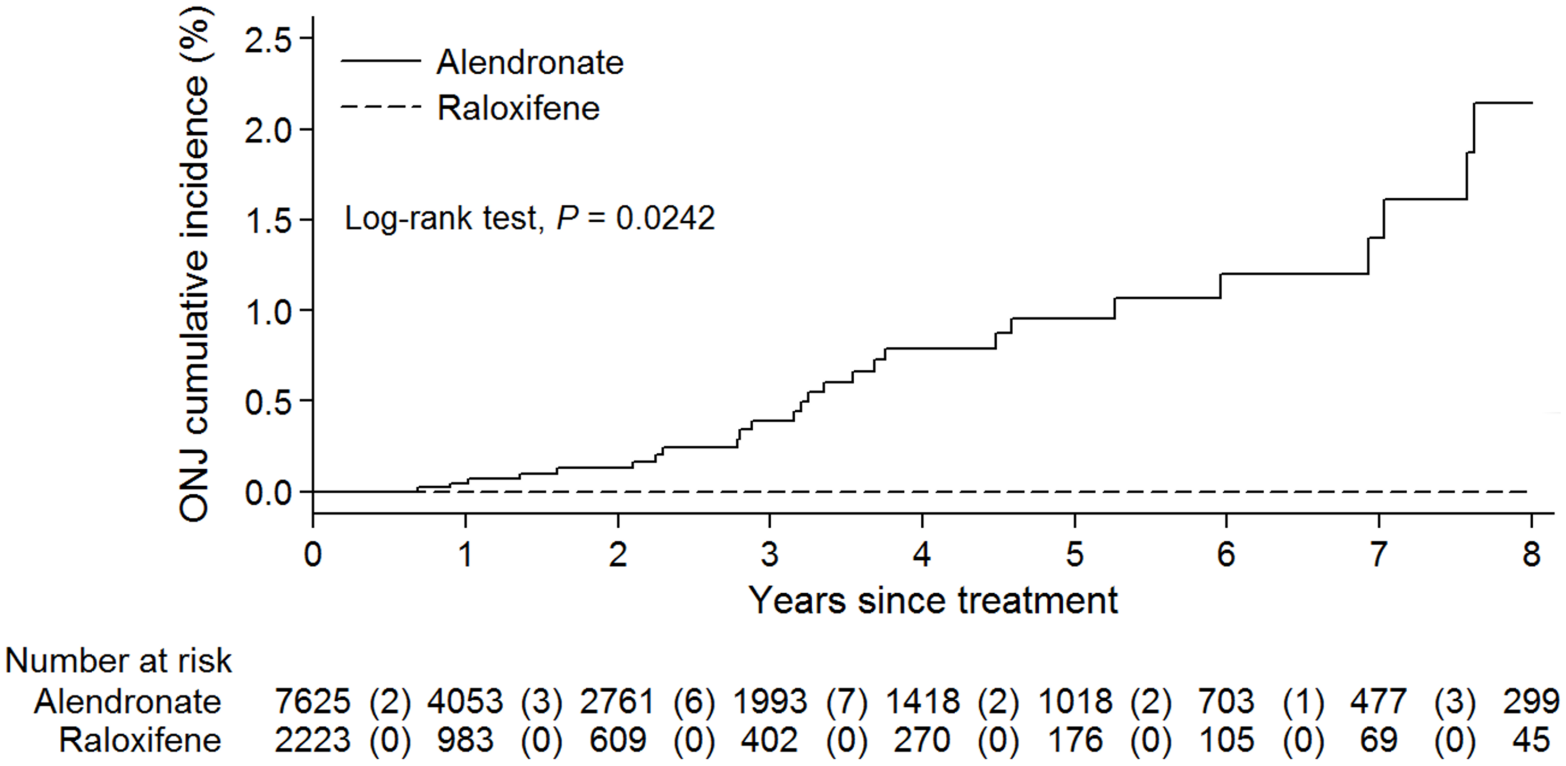
Time to the onset of ONJ in patients receiving alendronate or raloxifene. Numbers in parentheses indicate the number of ONJ cases at various times.

A total of 7,627 subjects fulfilled the requirements of the alendronate group while 2,223 individuals comprised the raloxifene group (Fig 1). Twenty-eight of 7,627 alendronate receivers had ONJ proven by diagnosis codes and subsequent operative therapies. Among these 28 patients, two cases had received other antiresorptive agents (one zoledronic acid and one ibandronate) between cessation of treatment and ONJ occurrence. Both patients were excluded and the remaining 7,625 patients (6,356 women, 1,269 men) were enrolled for analysis. Finally, a total of 26 ONJ cases (3 man and 23 women, S3 Table) were recognized in the alendronate group while no ONJ cases were noted in patients on raloxifene. The baseline characteristics before the use of alendronate or raloxifene are listed in S4 Table. Univariate analysis demonstrated significant differences in female patients (*P* < 0.001), age at the time of drug initiation (*P* = 0.0036), concomitant medical conditions of anemia (*P* = 0.004), and chronic kidney disease (*P* < 0.001). Because raloxifene was not licensed to treat male osteoporosis, it was not surprising that almost all the raloxifene users were women. Although the mean ages at which drug initiation occurred were significantly different between alendronate and raloxifene groups (73.75 versus 73.08, respectively; *P* = 0.0036, S3 Table), no difference was observed in female patients (73.09 versus 73.04 years, respectively; *P* = 0.826, raw data not included). The difference in age at drug initiation might result from a higher percentage of men in the alendronate group as the onset of osteoporosis is generally later among men than women. The remaining two differences might reflect drug preference (i.e., raloxifene over alendronate) for people with pre-existing chronic kidney disease. With respect to the ONJ risk following long-term alendronate or raloxifene therapy, the cumulative incidence of ONJ associated with alendronate ranged from 0.04% to 2.14% as the duration of treatment went from 1 year to 8 years, as opposed to no events for the patients on raloxifene (Fig 2).

### Tooth extractions increased the risk of ONJ

The frequencies of ONJ related to oral alendronate over a 13-year period were 0.36% in the women, 0.24% in the men, with an overall ONJ rate of 0.34%. The crude incidence of ONJ in individuals taking alendronate was approximately 262 per 100,000 persons per year. The characteristics of patients taking alendronate with and without ONJ are summarized in Table 1. There was no significant difference between affected cases and ONJ-free individuals except for duration of alendronate therapy (2.85 versus 1.39 years, *P* = 0.0001), cumulative dose of alendronate (5,113 versus 10,389 mg, *P* = 0.0001), proportion of subjects receiving TE within 1 year before the end date (42.3 versus 6.6%, *P* = 0.000) and rheumatoid arthritis (15.4 versus 4.3%, *P* = 0.024) based on univariate analysis. Among these 26 alendronate-related ONJ cases, 11 (42.3%) cases had preceding TE before they developed ONJ. There was a significantly greater proportion of ONJ occurrence among subjects taking alendronate with versus without antecedent TE in the prior year of end dates (2.16% versus 0.21%, *P* < 0.001), with a crude odds ratio of 10.46 (95% CI, 4.32–24.50, *P <* 0.001). These factors were further analyzed using a multiple logistic regression to predict the development of ONJ. Because of a close relationship between drug duration and the cumulative dose of alendronate as well as for easier ascertainment in clinical practice, drug duration rather than cumulative dose was chosen as an explanatory variable. The results demonstrated that longer drug exposure (≥ 3 years) (adjusted odds ratio, 3.00 [95% CI, 1.33–6.76]), rheumatoid arthritis (4.94 [1.64–14.90]), and preexisting TE (9.60 [4.33–21.29]) increased the risk of ONJ (Table 2).

**Table 1:** Clinical characteristics of patients taking alendronate with and without osteonecrosis of the jaw (ONJ).

Parameters^+^	Without ONJ (n = 7599)	With ONJ (n = 26)	*P*-value*
Female gender	6333 (83.3%)	23 (88.4%)	0.607
Age at drug initiation, mean (SD), years	73.75 (8.91)	73.51 (8.30)	0.8829
Duration of alendronate use, mean (SD), years	1.39 (1.69)	2.85 (1.61)	0.0001
Cumulative dose of alendronate, mean (SD), mg	5113.38(6219.67)	10389.46(5853.02)	0.0001
Diabetes mellitus	1883 (24.8%)	7 (26.9%)	0.821
Dyslipidemia	1748 (23.0%)	6 (23.1%)	1.000
Hypertension	3946 (51.9%)	10 (38.5%)	0.238
Rheumatoid arthritis	326 (4.3%)	4 (15.4%)	0.024
Ankylosing spondylitis	141 (1.9%)	1 (3.9%)	0.387
Diffuse diseases of connective tissue	233 (3.1%)	0	1.000
Chronic use of glucocorticoids^#^	369 (4.9%)	2 (7.7%)	0.363
Chronic use of methotrexate^#^	52 (0.7%)	0	1.000
Recent tooth extractions	498 (6.6%)	11 (42.3%)	0.000
Hypothyroidism	103 (1.4%)	0	1.000
Hyperthyroidism	98 (1.3%)	0	1.000
Anemia	771 (10.2%)	3 (11.5%)	0.743
Chronic kidney diseases	268 (3.5%)	0	1.000
Esophagitis or ulcer	698 (9.2%)	3 (11.5%)	0.728
Peptic ulcer	2050 (27.0%)	7 (26.9%)	1.000
Overall malignancy	697 (9.2%)	1 (3.9%)	0.508

ICD-9 codes included the following: diabetes mellitus, 250.xx; dyslipidemia, 272.x; hypertension, 401.x; rheumatoid arthritis, 714.xx; ankylosing spondylitis, 720.xx; diffuse diseases of connective tissue, 710.x; hypothyroidism, 243.x, 244.x; hyperthyroidism, 242.x; anemia, 280.x-285.x; chronic kidney disease, 585.x; esophagitis or ulcer, 530.1x, 530.2x; peptic ulcer, 531.xx-533.xx; overall malignancy, 150.x-159.x and 162.x-208.x

* *P*-value was calculated from a Fisher exact test or a *t* test with unequal variance for categorical or continuous variables, respectively

^+^ at the time of diagnosis of ONJ in affected cases or the last recorded dose in ONJ-free individuals

^#^ equivalent to 5 mg or more of prednisone daily or ≥7.5 mg/week of methotrexate for 3 months or longer

**Table 2:** Risk factors for the development of osteonecrosis of the jaw (ONJ) in patients receiving alendronate therapy.

Parameter	Adjusted Odds Ratio	95% CI	P*
Duration, ≥ 3 years *vs*. <3 years	3.00	1.33–6.76	0.008
Rheumatoid arthritis	4.94	1.64–14.90	0.005
Antecedent tooth extractions	9.60	4.33–21.29	0.000

* P-value was calculated from a multiple logistic regression analysis

Abbreviations: ONJ = osteonecrosis of the jaw; CI = confidence interval

### Tooth extractions increased the risk of ONJ independent of duration of alendronate therapy

**Fig 3:**
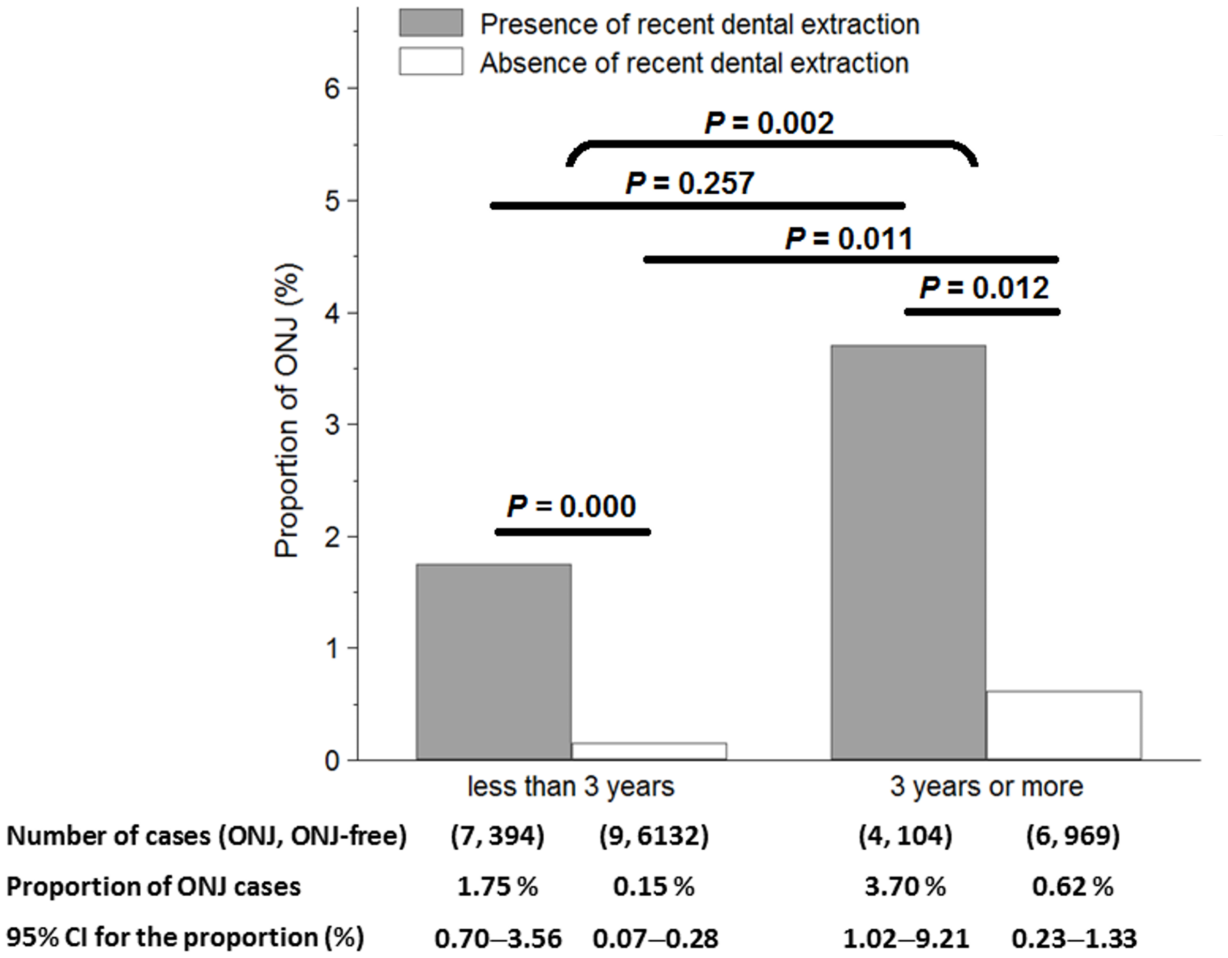
Tooth extraction increases the risk of ONJ independent of the duration of alendronate therapy. The overall prevalence of ONJ in osteoporotic subjects taking oral alendronate is estimated at 0.34% (26/7625) which rises to 2.16% (11/509) after recent tooth extraction. The difference in the proportions of ONJ between subjects with and without recent tooth extraction is computed using the Fisher’s exact method. Tooth extraction is significantly associated with increased risk for ONJ among osteoporotic patients taking alendronate for 3 years or more (*P* = 0.012) and in those with less than 3 years of use (*P* <0.001), independent of drug duration. In addition, there is a greater proportion of ONJ among patients with BP duration of 3 years or more versus less than 3 years of use (0.92% versus 0.24%, *P* = 0.002), supporting the influence of BP duration on ONJ occurrence.

To assess whether drug exposure duration influenced the relationship between ONJ occurrence and antecedent TE, the subjects were stratified into drug use for < 3 years or more. The rationale for this stratification was based on early literatures [15, 18] regarding oral BP use in osteoporotic patients which showed that a detectable risk for ONJ was generally not seen until an exposure of 2 to 3 years or more. TE was found to be significantly associated with increased risk for ONJ among osteoporotic patients taking alendronate no matter the drug duration (i.e., whether the duration was greater than or less than three years, Fig 3). In addition, the percentage of ONJ cases in the BP-treated osteoporotic subjects with less than 3 years of use (0.24%, 16/6526) as compared to 3 years or more (0.92%, 10/1073) was significantly lower (*P* = 0.002), indicating the positive influence of BP duration on ONJ development. A positive relationship was found (0.62% versus 0.15%, P = 0.011) among the subjects without preceding dentoalveolar procedures. However, among individuals exposed to recent dental extractions, prolonged BP duration led to a higher rate of ONJ cases, although this finding failed to reach significance (3.70% versus 1.75%, respectively; P = 0.257) compared to BP exposure of less than 3 years. The possible sources of uncertainty included small sample size and the covariate of TE—the strongest predictor for ONJ in the current study.

## Discussion

Earlier studies indicated that high-dose intravenous BP therapy used for a cancer indications was a contributing factor to the development of ONJ with a prevalence of > 5% [19, 20] after a few years. On the other hand, it is difficult to confirm a causal link between ONJ and the relatively lower doses of BP used for osteoporosis, since these problems can certainly also occur in the general population without BP use. Vestergaard et al [9] conducted a nationwide, register-based cohort study in Denmark to evaluate jaw-related events in BP users with nonusers and reported a hazard ratio of 3.15 (95% CI, 1.44 to 6.87) with alendronate use. Yamazaki and colleagues [5] completed a hospital-based cohort study in Japan which showed that oral bisphosphonates for osteoporosis were associated with an increased risk for ONJ with a relative risk 6.0 (95% CI, 1.3–26.1) after adjustment for confounding factors. In a study [4] carried out by our group in a hospital-based cohort of Taiwanese women treated with oral alendronate or raloxifene, the hazard ratio for ONJ occurrence with alendronate use was 7.42 (95% CI, 1.02 to 54.09) and the incidence rate was 283 per 100,000 person-years attributed to alendronate exposure. In the present nationwide cohort study, we reported ONJ in 26 of the 7,625 patients exposed to oral alendronate and in none of the 2223 patients exposed to raloxifene, thereby supporting an association between oral BP use for osteoporosis and ONJ. The estimated incidence rate of ONJ associated with alendronate was 262/100,000 person-years which was close to the risk reported in our earlier hospital-based study. These data supported the finding that oral BP therapy was associated with a 3.15 to 7.42-fold increased risk of ONJ regardless of treatment duration, age, gender, comorbidities or dentoalveolar surgery. It is important to determine accurately the ONJ incidence in BP-treated osteoporotic patients. Current estimates for ONJ associated with oral BP for osteoporosis in Western populations placed the incidence between 1.04 and 69 in 100,000 patient-treatment years [21]. In contrast, studies from Asian countries reported a wide range of ONJ incidence between 0.85 and 283 per 100,000 patient-years [4, 22–24]. The incidence of ONJ in BP-treated osteoporotic patients among various ethnic populations varies widely, suggesting the potential influence of oral hygiene or genetic background on the development of ONJ. On the other hand, by analyzing data from the Kaiser Permanente Northern California healthcare delivery system, among women older than 50 years who initiated oral BP therapy between 2002 and 2007, Lo et al. [25] reported that the rate of atypical femoral fractures was 7.2 per 100,000 person-years for white women and 64.2 per 100,000 person-years for Asian women and found that Asian women had a higher risk for atypical femoral fractures while taking a bisphosphonate compared with whites (hazard ratio of 6.57; 95% CI, 3.75–11.51) after adjusting for differences in bisphosphonate exposure and other risk factors. Collectively, these data suggest that compared with white women, Asians have a greater chance of experiencing ONJ and atypical femoral fracture while taking bisphosphonate therapy for osteoporosis. The mechanisms elucidating the ethnic differences in risks of BP-related dentoskeletal events require further investigation.

The association between ONJ and dentoalveolar procedures in cohorts of oncology patients treated with intravenous BP have been previously described, with a 16 to 33-fold increased risk for developing ONJ after TE [17]. However, the strength of the association between TE and ONJ occurrence in BP-treated osteoporotic patients remains poorly understood. In this current study, which enrolled osteoporotic patients taking oral alendronate, estimates for ONJ among participants without and with recent TE over a 13-year period were 0.21% and 2.16%, respectively. Multiple logistic regression analysis documented that an antecedent TE was the strongest contributor to ONJ occurrence (OR, 9.60 [4.33–21.29]) after adjusting for other potential confounders. This finding is consistent with previous evidence that TE was associated with ONJ development (OR, 6.6; 95% CI, 1.6–26.6) among patients without cancer [26]. More supportive evidence in needed to determine whether the risk of TE on ONJ development is different between subjects receiving intravenous BP for cancers versus osteoporotic patients taking BP orally.

Dentoalveolar procedures are not contraindicated in osteoporotic patients receiving oral BPs [17]. The AAOMS task Force on ONJ suggests discontinuation (drug holiday) of oral BPs for at least two months before invasive dental treatments in “at risk” patients with extended exposure history (>4 years), comorbidities, or concomitant glucocorticoids or antiangiogenic medications. On the other hand, no delay in planned oral surgery is considered for individuals who are exposed to an oral BP for less than four years and have no clinical risk factors. In this study, we found that TE was significantly associated with an increased risk of ONJ in patients with the BP duration exceeding three years as well as among subjects who have been treated with oral bisphosphonates for less than three years. It is advisable to recognize that invasive dental treatments can increase the risk for ONJ in all subjects receiving BP therapy, no matter the duration of exposure to BPs. Nevertheless, there is considerable controversy regarding whether or not a BP drug holiday for a certain period prior to invasive dental procedures is effective in reducing ONJ risk among BP-treated osteoporotic subjects [24, 27, 28].

Our study had several limitations including potential sources of observation bias which cannot be completely eliminated. One of the sources of such bias concerns the fact that patients who received dental procedures might have visited a dentist more frequently than others, leading to a higher probability of ONJ being detected at an early stage. In addition, the BP consumers, especially long-term users, are less likely to have TE because of concern about ONJ. The frequency of those dental procedures might have been intentionally reduced and the estimations may have been biased in the last treatment years of bisphosphonate therapy. The other source was the inconsistencies in case detection and diagnoses among health care professionals. We included only advanced ONJ with surgical indications to reduce such bias. Another major obstacle encountered in this research study was the lack of a specific diagnosis code for ONJ during the study period. To overcome a potential misclassification bias, we only enrolled patients whose diagnoses of osteomyelitis were made by the dentists or oral maxillofacial surgeons because most of the diagnosis codes were for inflammatory conditions involving the entire skeletal system, not exclusively the jaw bones. In this cohort analysis, the strategy to identify the ONJ cases required that all three criteria were met may lead to underestimation of ONJ incidence. In addition, dental implantation was not assessed in the current research because it was at one’s own expense and not included in NHIRD database. Although dental implant placement is also a potential trigger for ONJ occurrence, we feel that its effect on ONJ development could be explained by the preceding TE which is fully covered by our health insurance. In spite of these limitations, this national-wide cohort study provides a comprehensive basis for investigating the relationships among oral BP, TE, and the specific risks for ONJ in osteoporotic patients, which will increase the awareness concerning ONJ and serve as a source of information for the physicians treating osteoporotic patients.

In conclusion, the present study supports the association between ONJ and oral BP use with a relatively higher rate than that reported in Caucasian populations. TE was associated with a 9.6-fold increased risk of ONJ, independent of the duration of BP use. TE should be recognized as a contributing factor in the occurrence of ONJ irrespective of drug duration in osteoporotic patients receiving BP therapy.

## Supporting information

S1 Table Possible diagnosis codes for ONJ. (DOCX)

S2 Table Operative therapies to treat ONJ. (DOCX)

S3 Table Clinical characteristics of alendronate-related ONJ patients at the timing of first dental operation to treat ONJ. (DOCX)

S4 Table Comparisons of the baseline characteristics in patients taking alendronate or raloxifene. (DOCX)

## References

[1] Early Breast Cancer Trialists’ Collaborative G, Coleman R, Powles T, Paterson A, Gnant M, Anderson S, et al Adjuvant bisphosphonate treatment in early breast cancer: meta-analyses of individual patient data from randomised trials. Lancet. 2015;386(10001):1353–61. doi: 10.1016/S0140-6736(15)60908-4 . 26211824 10.1016/S0140-6736(15)60908-4

[2] Ben-Aharon I, Vidal L, Rizel S, Yerushalmi R, Shpilberg O, Sulkes A, et al Bisphosphonates in the adjuvant setting of breast cancer therapy—effect on survival: a systematic review and meta-analysis. PloS one. 2013;8(8):e70044 doi: 10.1371/journal.pone.0070044 . 23990894 10.1371/journal.pone.0070044 PMC3753308

[3] Mhaskar R, Redzepovic J, Wheatley K, Clark OA, Miladinovic B, Glasmacher A, et al Bisphosphonates in multiple myeloma: a network meta-analysis. Cochrane Database Syst Rev. 2012;5:CD003188 doi: 10.1002/14651858.CD003188.pub3 . 22592688 10.1002/14651858.CD003188.pub3

[4] Chiu WY, Chien JY, Yang WS, Juang JM, Lee JJ, Tsai KS. The risk of osteonecrosis of the jaws in Taiwanese osteoporotic patients treated with oral alendronate or raloxifene. J Clin Endocrinol Metab. 2014;99(8):2729–35. doi: 10.1210/jc.2013-4119 . 24758181 10.1210/jc.2013-4119

[5] Yamazaki T, Yamori M, Yamamoto K, Saito K, Asai K, Sumi E, et al Risk of osteomyelitis of the jaw induced by oral bisphosphonates in patients taking medications for osteoporosis: a hospital-based cohort study in Japan. Bone. 2012;51(5):882–7. doi: 10.1016/j.bone.2012.08.115 . 22917933 10.1016/j.bone.2012.08.115

[6] Hong JW, Nam W, Cha IH, Chung SW, Choi HS, Kim KM, et al Oral bisphosphonate-related osteonecrosis of the jaw: the first report in Asia. Osteoporos Int. 2010;21(5):847–53. doi: 10.1007/s00198-009-1024-9 . 19633881 10.1007/s00198-009-1024-9

[7] Lo JC, O’Ryan F, Yang J, Hararah MK, Gonzalez JR, Gordon N, et al Oral health considerations in older women receiving oral bisphosphonate therapy. Journal of the American Geriatrics Society. 2011;59(5):916–22. doi: 10.1111/j.1532-5415.2011.03371.x . 21539523 10.1111/j.1532-5415.2011.03371.x

[8] Hansen PJ, Knitschke M, Draenert FG, Irle S, Neff A. Incidence of bisphosphonate-related osteonecrosis of the jaws (BRONJ) in patients taking bisphosphonates for osteoporosis treatment—a grossly underestimated risk? Clin Oral Investig. 2013;17(8):1829–37. doi: 10.1007/s00784-012-0873-3 . 23114879 10.1007/s00784-012-0873-3

[9] Vestergaard P, Schwartz K, Rejnmark L, Mosekilde L, Pinholt EM. Oral bisphosphonate use increases the risk for inflammatory jaw disease: a cohort study. J Oral Maxillofac Surg. 2012;70(4):821–9. doi: 10.1016/j.joms.2011.02.093 . 21764202 10.1016/j.joms.2011.02.093

[10] Yamazaki T, Yamori M, Tanaka S, Yamamoto K, Sumi E, Nishimoto-Sano M, et al Risk factors and indices of osteomyelitis of the jaw in osteoporosis patients: results from a hospital-based cohort study in Japan. PloS one. 2013;8(11):e79376 doi: 10.1371/journal.pone.0079376 . 24223935 10.1371/journal.pone.0079376 PMC3815193

[11] Saad F, Brown JE, Van Poznak C, Ibrahim T, Stemmer SM, Stopeck AT, et al Incidence, risk factors, and outcomes of osteonecrosis of the jaw: integrated analysis from three blinded active-controlled phase III trials in cancer patients with bone metastases. Ann Oncol. 2012;23(5):1341–7. doi: 10.1093/annonc/mdr435 . 21986094 10.1093/annonc/mdr435

[12] Vahtsevanos K, Kyrgidis A, Verrou E, Katodritou E, Triaridis S, Andreadis CG, et al Longitudinal cohort study of risk factors in cancer patients of bisphosphonate-related osteonecrosis of the jaw. J Clin Oncol. 2009;27(32):5356–62. doi: 10.1200/JCO.2009.21.9584 . 19805682 10.1200/JCO.2009.21.9584

[13] Fehm T, Beck V, Banys M, Lipp HP, Hairass M, Reinert S, et al Bisphosphonate-induced osteonecrosis of the jaw (ONJ): Incidence and risk factors in patients with breast cancer and gynecological malignancies. Gynecol Oncol. 2009;112(3):605–9. doi: 10.1016/j.ygyno.2008.11.029 . 19136147 10.1016/j.ygyno.2008.11.029

[14] Lee JJ, Cheng SJ, Wang JJ, Chiang CP, Chang HH, Chen HM, et al Factors predicting the prognosis of oral alendronate-related osteonecrosis of the jaws: a 4-year cohort study. Head & neck. 2013;35(12):1787–95. doi: 10.1002/hed.23235 . 23508560 10.1002/hed.23235

[15] Lo JC, O’Ryan FS, Gordon NP, Yang J, Hui RL, Martin D, et al Prevalence of osteonecrosis of the jaw in patients with oral bisphosphonate exposure. J Oral Maxillofac Surg. 2010;68(2):243–53. doi: 10.1016/j.joms.2009.03.050 . 19772941 10.1016/j.joms.2009.03.050 PMC10159647

[16] Howie RN, Borke JL, Kurago Z, Daoudi A, Cray J, Zakhary IE, et al A Model for Osteonecrosis of the Jaw with Zoledronate Treatment following Repeated Major Trauma. PloS one. 2015;10(7):e0132520 doi: 10.1371/journal.pone.0132520 . 26186665 10.1371/journal.pone.0132520 PMC4505856

[17] Ruggiero SL, Dodson TB, Fantasia J, Goodday R, Aghaloo T, Mehrotra B, et al American Association of Oral and Maxillofacial Surgeons position paper on medication-related osteonecrosis of the jaw—2014 update. J Oral Maxillofac Surg. 2014;72(10):1938–56. doi: 10.1016/j.joms.2014.04.031 . 25234529 10.1016/j.joms.2014.04.031

[18] Palaska PK, Cartsos V, Zavras AI. Bisphosphonates and time to osteonecrosis development. Oncologist. 2009;14(11):1154–66. doi: 10.1634/theoncologist.2009-0115 . 19897878 10.1634/theoncologist.2009-0115

[19] Bamias A, Kastritis E, Bamia C, Moulopoulos LA, Melakopoulos I, Bozas G, et al Osteonecrosis of the jaw in cancer after treatment with bisphosphonates: incidence and risk factors. J Clin Oncol. 2005;23(34):8580–7. doi: 10.1200/JCO.2005.02.8670 . 16314620 10.1200/JCO.2005.02.8670

[20] Thumbigere-Math V, Tu L, Huckabay S, Dudek AZ, Lunos S, Basi DL, et al A retrospective study evaluating frequency and risk factors of osteonecrosis of the jaw in 576 cancer patients receiving intravenous bisphosphonates. Am J Clin Oncol. 2012;35(4):386–92. . 22561331 10.1097/COC.0b013e3182155fcb

[21] Khan AA, Morrison A, Hanley DA, Felsenberg D, McCauley LK, O’Ryan F, et al Diagnosis and management of osteonecrosis of the jaw: a systematic review and international consensus. J Bone Miner Res. 2015;30(1):3–23. doi: 10.1002/jbmr.2405 . 25414052 10.1002/jbmr.2405

[22] Yoneda T, Hagino H, Sugimoto T, Ohta H, Takahashi S, Soen S, et al Bisphosphonate-related osteonecrosis of the jaw: position paper from the Allied Task Force Committee of Japanese Society for Bone and Mineral Research, Japan Osteoporosis Society, Japanese Society of Periodontology, Japanese Society for Oral and Maxillofacial Radiology, and Japanese Society of Oral and Maxillofacial Surgeons. Journal of bone and mineral metabolism. 2010;28(4):365–83. doi: 10.1007/s00774-010-0162-7 . 20333419 10.1007/s00774-010-0162-7

[23] Kwok T, Choy TK, Kwok WL. Prevalence of bisphosphonate-related osteonecrosis of the jaw in Hong Kong. Hong Kong Med J. 2016;22 Suppl 2:S46–7. . 26908345

[24] Japanese Allied Committee on Osteonecrosis of the J, Yoneda T, Hagino H, Sugimoto T, Ohta H, Takahashi S, et al Antiresorptive agent-related osteonecrosis of the jaw: Position Paper 2017 of the Japanese Allied Committee on Osteonecrosis of the Jaw. Journal of bone and mineral metabolism. 2017;35(1):6–19. doi: 10.1007/s00774-016-0810-7 . 28035494 10.1007/s00774-016-0810-7

[25] Lo JC, Hui RL, Grimsrud CD, Chandra M, Neugebauer RS, Gonzalez JR, et al The association of race/ethnicity and risk of atypical femur fracture among older women receiving oral bisphosphonate therapy. Bone. 2016;85:142–7. doi: 10.1016/j.bone.2016.01.002 . 26769007 10.1016/j.bone.2016.01.002 PMC5108728

[26] Barasch A, Cunha-Cruz J, Curro FA, Hujoel P, Sung AH, Vena D, et al Risk factors for osteonecrosis of the jaws: a case-control study from the CONDOR dental PBRN. Journal of dental research. 2011;90(4):439–44. doi: 10.1177/0022034510397196 . 21317246 10.1177/0022034510397196 PMC3144129

[27] Hasegawa T, Kawakita A, Ueda N, Funahara R, Tachibana A, Kobayashi M, et al A multicenter retrospective study of the risk factors associated with medication-related osteonecrosis of the jaw after tooth extraction in patients receiving oral bisphosphonate therapy: can primary wound closure and a drug holiday really prevent MRONJ? Osteoporos Int. 2017;28(8):2465–73. doi: 10.1007/s00198-017-4063-7 . 28451732 10.1007/s00198-017-4063-7

[28] Hasegawa T, Ri S, Umeda M, Komatsubara H, Kobayashi M, Shigeta T, et al The observational study of delayed wound healing after tooth extraction in patients receiving oral bisphosphonate therapy. J Craniomaxillofac Surg. 2013;41(7):558–63. doi: 10.1016/j.jcms.2012.11.023 . 23332469 10.1016/j.jcms.2012.11.023

