# Supplementary material for: The influence of alendronate and tooth extraction on the incidence of osteonecrosis of the jaw among osteoporotic subjects
Source: PLoS One. 2018 Apr 25;13(4):e0196419. doi: 10.1371/journal.pone.0196419 (PMC5918995; doi:10.1371/journal.pone.0196419)
Supplement: S1 Table — (DOCX) [file pone.0196419.s001.docx]

# S1 Table. Possible diagnosis codes for ONJ

| **ICD-9-CM Diagnosis Code** | **Description** |
| --- | --- |
| 526.4 | Inflammatory conditions of jaw |
| 526.5 | Alveolitis of jaw |
| 730.00 | Acute osteomyelitis, site unspecified |
| 730.08 | Acute osteomyelitis involving other specified sites |
| 730.1 | chronic osteomyelitis |
| 730.10 | Chronic osteomyelitis, site unspecified |
| 730.18 | Chronic osteomyelitis involving other specified sites |
| 730.20 | Unspecified osteomyelitis, site unspecified |
| 730.28 | Unspecified osteomyelitis, other specified sites |
| 733.4 | Aseptic necrosis of bone |
| 733.40 | Aseptic necrosis of bone, site unspecified |
| 733.45 | Osteonecrosis of the jaws |
| 733.49 | Aseptic necrosis of other bone sites |
