# Supplementary material for: The influence of alendronate and tooth extraction on the incidence of osteonecrosis of the jaw among osteoporotic subjects
Source: PLoS One. 2018 Apr 25;13(4):e0196419. doi: 10.1371/journal.pone.0196419 (PMC5918995; doi:10.1371/journal.pone.0196419)
Supplement: S2 Table — (DOCX) [file pone.0196419.s002.docx]

# S2 Table. Operative therapies to treat ONJ

| **Code** | **Procedures** |
| --- | --- |
| 64005B | Sequestrectomy or saucerization & debridement for osteomyelitis |
| 65009B | Caldwell Luc’s operation, unilateral |
| 65038B | Maxillectomy, partial |
| 65039B | Maxillectomy, total |
| 92025B | Sequestrectomy, simple case under 1/3 arch |
| 92026B | Sequestrectomy, complicated case more than 1/3 arch |
| 92204B | Saucerization and sequestrectomy |
| 92205B | Saucerization |
| 92211B | Resection of the jaw (each), marginal |
| 92212B | Resection of the jaw (each), partial |
| 92213B | Resection of the jaw (each), hemi-resection |
