# Supplementary material for: The influence of alendronate and tooth extraction on the incidence of osteonecrosis of the jaw among osteoporotic subjects
Source: PLoS One. 2018 Apr 25;13(4):e0196419. doi: 10.1371/journal.pone.0196419 (PMC5918995; doi:10.1371/journal.pone.0196419)
Supplement: S3 Table — (DOCX) [file pone.0196419.s003.docx]

**S3 Table. Clinical characteristics of alendronate-related ONJ patients at the timing of first dental operation to treat ONJ**

| **ID** | **Sex** | **Age** | **Coday** | **Cumdose** | **Exdent** | **ONJ_icd9** | **ONJ_OP** | **DM** | **HTN** | **Lipid** | **RA** | **AS** | **DCT** | **GC** | **MTX** | **HypoT** | **HyperT** | **Anem** | **CKD** | **Esop** | **Pept** | **OverCA** |
| --- | --- | --- | --- | --- | --- | --- | --- | --- | --- | --- | --- | --- | --- | --- | --- | --- | --- | --- | --- | --- | --- | --- |
| 1 | M | 83.5784 | 2.28611 | 8350 | 1 | 526.4/733.49 | 64005B | 0 | 1 | 1 | 0 | 0 | 0 | 0 | 0 | 0 | 0 | 0 | 0 | 0 | 0 | 0 |
| 2 | F | 64.627 | 0.97741 | 3570 | 0 | 526.4 | 64005B | 0 | 1 | 0 | 0 | 0 | 0 | 0 | 0 | 0 | 0 | 0 | 0 | 0 | 0 | 0 |
| 3 | F | 66.7132 | 3.06639 | 11200 | 0 | 730.10 | 92025B | 1 | 0 | 0 | 0 | 1 | 0 | 0 | 0 | 0 | 0 | 0 | 0 | 0 | 1 | 0 |
| 4 | F | 66.4531 | 2.76249 | 10090 | 0 | 526.5 | 92025B | 1 | 1 | 0 | 1 | 0 | 0 | 0 | 0 | 0 | 0 | 0 | 0 | 0 | 0 | 0 |
| 5 | M | 83.3019 | 2.10267 | 7830 | 1 | 526.5 | 92025B | 0 | 0 | 0 | 0 | 0 | 0 | 0 | 0 | 0 | 0 | 1 | 0 | 0 | 0 | 0 |
| 6 | F | 84.7529 | 3.22245 | 12730 | 0 | 526.4/730.20 | 64005B | 0 | 1 | 0 | 0 | 0 | 0 | 0 | 0 | 0 | 0 | 0 | 0 | 1 | 1 | 1 |
| 7 | F | 91.5181 | 6.49966 | 23740 | 1 | 526.4 | 92025B | 0 | 0 | 0 | 1 | 0 | 0 | 0 | 0 | 0 | 0 | 0 | 0 | 0 | 0 | 0 |
| 8 | F | 73.2758 | 4.91171 | 17950 | 0 | 526.5 | 92025B | 0 | 0 | 0 | 0 | 0 | 0 | 0 | 0 | 0 | 0 | 0 | 0 | 0 | 0 | 0 |
| 9 | F | 76.6543 | 4.30664 | 15780 | 1 | 730.28 | 92025B | 0 | 0 | 1 | 0 | 0 | 0 | 0 | 0 | 0 | 0 | 0 | 0 | 0 | 0 | 0 |
| 10 | F | 91.7645 | 0.68994 | 2520 | 0 | 526.4 | 92026B | 0 | 1 | 0 | 0 | 0 | 0 | 0 | 0 | 0 | 0 | 1 | 0 | 0 | 0 | 0 |
| 11 | F | 69.3717 | 1.34155 | 4900 | 0 | 526.4 | 92025B | 0 | 0 | 0 | 0 | 0 | 0 | 0 | 0 | 0 | 0 | 0 | 0 | 0 | 0 | 0 |
| 12 | M | 83.499 | 1.99316 | 7280 | 1 | 526.4 | 64005B | 1 | 1 | 0 | 0 | 0 | 0 | 0 | 0 | 0 | 0 | 0 | 0 | 0 | 0 | 0 |
| 13 | F | 64.2793 | 3.90144 | 13496 | 0 | 526.4 | 92205B | 0 | 0 | 1 | 0 | 0 | 0 | 1 | 0 | 0 | 0 | 0 | 0 | 1 | 1 | 0 |
| 14 | F | 83.4415 | 2.2998 | 8400 | 0 | 526.4/526.5/730.18/730.28 | 92025B | 0 | 0 | 0 | 0 | 0 | 0 | 0 | 0 | 0 | 0 | 0 | 0 | 0 | 0 | 0 |
| 15 | F | 66.653 | 2.45311 | 8960 | 1 | 526.4 | 92025B | 0 | 0 | 1 | 0 | 0 | 0 | 0 | 0 | 0 | 0 | 0 | 0 | 0 | 0 | 0 |
| 16 | F | 80.0821 | 4.06297 | 14840 | 0 | 526.5/730.2 | 64005B | 1 | 0 | 1 | 0 | 0 | 0 | 0 | 0 | 0 | 0 | 0 | 0 | 0 | 0 | 0 |
| 17 | F | 70.0479 | 4.13963 | 15120 | 1 | 526.4 | 92025B | 0 | 1 | 0 | 1 | 0 | 0 | 1 | 0 | 0 | 0 | 0 | 0 | 0 | 0 | 0 |
| 18 | F | 73.065 | 2.65845 | 9710 | 1 | 526.4 | 92025B | 1 | 1 | 0 | 0 | 0 | 0 | 0 | 0 | 0 | 0 | 0 | 0 | 0 | 0 | 0 |
| 19 | F | 80.386 | 7.01164 | 25170 | 0 | 526.4 | 92025B | 0 | 0 | 0 | 1 | 0 | 0 | 0 | 0 | 0 | 0 | 0 | 0 | 0 | 0 | 0 |
| 20 | F | 72.4572 | 2.0835 | 7610 | 1 | 730.18 | 92204B | 0 | 0 | 0 | 0 | 0 | 0 | 0 | 0 | 0 | 0 | 0 | 0 | 0 | 0 | 0 |
| 21 | F | 77.8179 | 1.5332 | 5600 | 0 | 526.4 | 64005B | 0 | 1 | 0 | 0 | 0 | 0 | 0 | 0 | 0 | 0 | 0 | 0 | 0 | 0 | 0 |
| 22 | F | 79.2361 | 3.37851 | 12390 | 1 | 526.4 | 64005B | 1 | 0 | 0 | 0 | 0 | 0 | 0 | 0 | 0 | 0 | 0 | 0 | 0 | 1 | 0 |
| 23 | F | 78.9651 | 1.1937 | 4200 | 0 | 526.4 | 92025B | 0 | 0 | 0 | 0 | 0 | 0 | 0 | 0 | 0 | 0 | 0 | 0 | 0 | 1 | 0 |
| 24 | F | 76.1862 | 1.80151 | 6580 | 1 | 526.4/526.5 | 65038B | 1 | 1 | 0 | 0 | 0 | 0 | 0 | 0 | 0 | 0 | 1 | 0 | 1 | 1 | 0 |
| 25 | F | 78.768 | 0.68994 | 2520 | 0 | 526.4 | 92025B | 0 | 0 | 1 | 0 | 0 | 0 | 0 | 0 | 0 | 0 | 0 | 0 | 0 | 0 | 0 |
| 26 | F | 88.3723 | 2.6256 | 9590 | 0 | 526.4 | 92204B | 0 | 0 | 0 | 0 | 0 | 0 | 0 | 0 | 0 | 0 | 0 | 0 | 0 | 1 | 0 |

**Description of variables**: ID, patient; Sex, gender; Age, age at endpoint (years); Coday, duration of drug exposure (years); Cumdose, cumulative dose of drug (mg); Exdent, tooth extractoin was ascertained within 1 year before the endpoint; ONJ_icd9, diagnosis codes for ONJ (see S1 Table); ONJ_OP, dental operation for ONJ (see S2 Table); DM, diabetes mellitus (ICD9 code 250.xx); HTN, hypertension (ICD9 code 401.x); Lipid, dyslipidemia (ICD9 code 272.x); RA, rheumatoid arthritis (ICD9 code 714.xx); AS, ankylosing spondylitis (ICD9 code 720.xx); DCT, diffuse connective tissue disease (ICD9 code 710.x); GC, chronic steroid use (equivalent to 5 mg or more of prednisone daily for 3 months); MTX, chronic methotrexate use (≧7.5 mg/week of methotrexat for 3 months); HypoT, hypothyroidism (ICD9 codes 243.x, 244.x); HyperT, hyperthyroidism (ICD9 code 242.x); Anem, anemia (ICD9 codes 280.x-285.x); CKD, chronic kidney disease (ICD9 code 585.x); Esop, esophagitis or ulcer (ICD9 codes 530.1x, 530.2x); Pept, peptic ulcer (ICD9 codes 531.xx-533.xx); OverCA, overall malignancy (ICD9 codes 150.x-159.x and 162.x-208.x)
