# Supplementary material for: The influence of alendronate and tooth extraction on the incidence of osteonecrosis of the jaw among osteoporotic subjects
Source: PLoS One. 2018 Apr 25;13(4):e0196419. doi: 10.1371/journal.pone.0196419 (PMC5918995; doi:10.1371/journal.pone.0196419)
Supplement: S4 Table — (DOCX) [file pone.0196419.s004.docx]

**S4 Table. Comparisons of the baseline characteristics in patients taking alendronate or raloxifene**

| **Parameters^+^** | **Alendronate (n=7625)** | **Raloxifene (n=2223)** | ***P*-value*** |
| --- | --- | --- | --- |
| Female | 6356 (83.4 %) | 2206 (99.2 %) | 0.000 |
| Age at drug initiation, mean (SD), years | 73.75 (8.91) | 73.08 (9.66) | 0.0036 |
| Diabetes mellitus | 1780 (23.3 %) | 552 (24.8 %) | 0.148 |
| Dyslipidemia | 1714 (22.5 %) | 509 (22.9 %) | 0.687 |
| Hypertension | 3771 (49.5 %) | 1073 (48.3 %) | 0.335 |
| Rheumatoid arthritis | 329 (4.3 %) | 112 (5.0 %) | 0.146 |
| Ankylosing spondylitis | 166 (2.2 %) | 36 (1.6 %) | 0.107 |
| Diffuse diseases of connective tissue | 208 (2.7 %) | 63 (2.8 %) | 0.769 |
| Chronic use of glucocorticoids^#^ | 337 (4.4 %) | 81 (3.6 %) | 0.120 |
| Chronic use of methotrexate^#^ | 45 (0.6 %) | 14 (0.6 %) | 0.876 |
| Hypothyroidism | 84 (1.1 %) | 24 (1.1 %) | 1.000 |
| Hyperthyroidism | 97 (1.3 %) | 34 (1.5 %) | 0.345 |
| Anemia | 674 (8.8 %) | 242 (10.9 %) | 0.004 |
| Chronic kidney diseases | 177 (2.3 %) | 110 (5.0 %) | 0.000 |
| Esophagitis or ulcer | 555 (7.3 %) | 156 (7.0 %) | 0.710 |
| Peptic ulcer | 1955 (25.6 %) | 592 (26.6 %) | 0.349 |
| Overall malignancy | 551 (7.2 %) | 151 (6.8 %) | 0.512 |

ICD-9 codes included the following: diabetes mellitus, 250.xx; dyslipidemia, 272.x; hypertension, 401.x; rheumatoid arthritis, 714.xx; ankylosing spondylitis, 720.xx; diffuse diseases of connective tissue, 710.x; hypothyroidism, 243.x, 244.x; hyperthyroidism, 242.x; anemia, 280.x-285.x; chronic kidney disease, 585.x; esophagitis or ulcer, 530.1x, 530.2x; peptic ulcer, 531.xx-533.xx; overall malignancy, 150.x-159.x and 162.x-208.x

*^*^ P*-value was calculated from a Fisher’s exact test or a *t* test with unequal variance for categorical or continuous variables, respectively

^+^ at the time of enrollment

^#^ equivalent to 5 mg or more of prednisone daily or ≥7.5 mg/week of methotrexate for 3 months or longer
